# Dietary patterns and colorectal cancer risk in Zimbabwe: A population based case-control study

**DOI:** 10.1016/j.canep.2018.09.005

**Published:** 2018-12

**Authors:** Leolin Katsidzira, Ria Laubscher, Innocent T. Gangaidzo, Rina Swart, Rudo Makunike-Mutasa, Tadios Manyanga, Sandie Thomson, Raj Ramesar, Jonathan A. Matenga, Simbarashe Rusakaniko

**Affiliations:** a Department of Medicine, College of Health Sciences, University of Zimbabwe, PO Box A178, Avondale, Harare, Zimbabwe; b Biostatistics Unit, South African Medical Research Council, PO Box 19070, Tygerberg, Cape Town, 7505, South Africa; c Department of Dietetics and Nutrition, University of Western Cape, Bellville, 7535, Western Cape, South Africa; d Department of Histopathology, College of Health Sciences, University of Zimbabwe, PO Box A178, Avondale, Harare, Zimbabwe; e Division of Gastroenterology, Department of Medicine, University of Cape Town, Groote Schuur Hospital, Observatory, 7925, Cape Town, South Africa; f MRC/UCT Research Unit for Genomic and Precision Medicine, Division of Human Genetics, Institute of Infectious Diseases and Molecular Medicine, Faculty of Health Sciences, University of Cape Town, Observatory, 7925, South Africa; g Department of Community Medicine, College of Health Sciences, University of Zimbabwe, PO Box A178, Avondale, Harare, Zimbabwe

**Keywords:** OR, odds ratio, CI, confidence interval, FAO, Food and Agriculture Organization of the United Nations, BMI, body mass index, Colorectal neoplasms, Africa South of the Sahara, Risk factors, Incidence, Diet, Zimbabwe

## Abstract

**Background:**

The rising incidence of colorectal cancer in sub-Saharan Africa may be partly caused by changing dietary patterns. We sought to establish the association between dietary patterns and colorectal cancer in Zimbabwe.

**Methods:**

One hundred colorectal cancer cases and 200 community-based controls were recruited. Data were collected using a food frequency questionnaire, and dietary patterns derived by principal component analysis. Generalised linear and logistic regression models were used to assess the associations between dietary patterns, participant characteristics and colorectal cancer.

**Results:**

Three main dietary patterns were identified: traditional African, urbanised and processed food. The traditional African diet appeared protective against colorectal cancer (Odds Ratio (OR) 0.35; 95% Confidence Interval (CI), 0.21 – 0.58), which had no association with the urban (OR 0.68; 95% CI, 0.43–1.08), or processed food (OR 0.91; 0.58–1.41) patterns. The traditional African diet was associated with rural domicile, (OR 1.26; 95% CI, 1.00–1.59), and a low income (OR1.48; 95% CI, 1.06–2.08). The urbanised diet was associated with urban domicile (OR 1.70; 95% CI, 1.38–2.10), secondary (OR 1.30; 95% CI, 1.07–1.59) or tertiary education (OR 1.48; 95% CI, 1.11–1.97), and monthly incomes of $201–500 (OR 1.30; 95% CI, 1.05–1.62), and the processed food pattern with tertiary education (OR 1.42; 95% CI, 1.05–1.92), and income >$1000/month (OR 1.48; 95% CI, 1.02–2.15).

**Conclusion:**

A shift away from protective, traditional African dietary patterns may partly explain the rising incidence of colorectal cancer in sub-Saharan Africa.

## Highlights

• Three main dietary patterns in Zimbabwe; traditional African, urban and processed foods.
• Traditional African diet associated with a reduced risk of colorectal cancer.
• No association between colorectal cancer and the urban or processed food patterns.

## 1 Introduction

Colorectal cancer is the 3rd most common cancer globally, and the 6th most common in Africa [1,2]. The age standardised incidence of colorectal cancer in sub-Saharan Africa per 100,000 ranges from 3.1 in men and 2.9 in women in Malawi, to 14.9 in men and 14.2 in women in Zimbabwe [3]. There has been a gradual temporal increase in the incidence of colorectal cancer in Zimbabwe over the past two decades [4,5]. Despite this increase, the incidence of colorectal cancer is still significantly lower than in the developed world, where the rate is approximately three times higher [6]. An increase in the burden of colorectal cancer has also been described in other parts of sub-Saharan Africa, including Mozambique, Kenya and Nigeria [[7], [8], [9]]. This rising incidence has been attributed to changes in diet and lifestyle, and to improved diagnosis [10]. However, the role of dietary changes in the rising incidence of colorectal cancer is not supported by solid empirical data.

It is generally accepted that dietary factors account for the majority of sporadic colorectal cancers [11]. This link is strongest for a high intake of red meat and processed meat products, which are now classified by the International Agency for Research on Cancer (IARC) as ‘probably carcinogenic’, and ‘carcinogenic’ to humans respectively [12]. Other dietary factors associated with an increased risk of colorectal cancer include a high intake of alcohol, animal fat and sugar [11]. In contrast, a high intake of dietary fibre, non-starchy vegetables, fruits, milk, calcium, and vitamin D is associated with a reduced risk [11]. However, the causative role of diet on colorectal cancer is more complex than implied by the degree of risk attributed to individual components. There is considerable interplay between the different nutritive and non-nutritive components, underscoring the importance of considering the overall dietary pattern. Furthermore, the overall diet has a major impact on gut microbiota composition and function, which plays a key role in the development of colorectal cancer [13].

Nonetheless, previous studies on the effect of diet in sub-Saharan Africa focussed on the putative protective role of individual constituents [10]. Another limitation of earlier studies was the absence of individuals with colorectal cancer as the comparator. The studies invariably recruited healthy people only, who were all assumed to have a uniformly low risk. There is need to evaluate the role of dietary patterns on the risk of colorectal cancer in sub-Saharan Africa using neoplasia as the end-point. This is particularly relevant now, given the on-going changes in dietary practices in the region. There is rising intake of meat, processed animal products and high energy foods, and decreasing intake of traditional grains and plants [14]. Therefore, we sought to establish whether there is an association between dietary patterns and colorectal cancer risk in an African population in Zimbabwe.

## 2 Materials and methods

### 2.1 Study population

The study design and population have been described previously [15]. Briefly, a population based case-control study of adult black Zimbabweans with colorectal cancer and community based controls was carried out. The cases were recruited through all the private and public tertiary level clinical, endoscopic and pathology services within Harare. These provide tertiary referral services for the northern two thirds of Zimbabwe. Only histologically confirmed cases of colorectal cancer were considered for inclusion, and they were recruited within six months of diagnosis. Individuals with recurrent colorectal cancer, or cognitive impairment were excluded. The controls were selected from households in the areas where the cases ordinarily lived, using enumeration maps from the 2012 Zimbabwe national census. Two controls were selected for each case, and they were matched for sex, and age to within 5 years. Ethical approval was obtained from the institutional review committees of the University of Zimbabwe College of Health Sciences and the Medical Research Council of Zimbabwe.

### 2.2 Data collection

Informed consent was obtained from all participants, who were then interviewed using a validated semi-quantitative food frequency questionnaire containing [16]. The questionnaire contained 110 items, after adjustments to account for foods suspected to represent distinct dietary patterns, or to affect the risk of colorectal cancer. Data on medical history, demographic and socio-economic characteristics was also collected. This included age, sex, domicile at different stages of life, level of education, employment status, income, use of non-steroidal anti-inflammatory drugs, smoking, alcohol consumption, and family history of all cancers, and of colorectal cancer in particular. For the cases, data on the tumour location was obtained from pathology reports and clinical notes. The anatomic sites were defined as follows: proximal colon (caecum to transverse colon), distal colon (splenic flexure to sigmoid colon), and rectum (recto-sigmoid junction and rectum). The controls were interviewed at home, and the cases were seen in hospital or at home.

### 2.3 Statistical analysis

It was estimated that a sample size of 100 cases and 200 controls would give at least 80% power to detect an absolute difference of 20% in exposure rates using a two-sided α of 0.05. Baseline demographic and clinical characteristics were compared between cases and controls using a chi-squared or fisher’s exact test for categorical variables, and a student *t*-test for continuous variables. A p value of <0.05 was regarded as statistically significant. The 110 different foods were grouped *a posteriori* into 31 groups using the dietary diversity score instrument from the Food and Agriculture Organization of the United Nations (FAO) [17], with adjustments to reflect Zimbabwean culinary practice. Principal component analysis was used to derive dietary patterns from these food groups. An eigenvalue >1 and scree plots were used to determine the factors responsible for most of the variability. These factors were retained, and orthogonal rotation was performed to enhance interpretability. Rotated factors loadings greater than 0.35 were used to select meaningful associations of food groups. The Kaiser-Meyer-Olkin test was used to assess sampling adequacy. The retained factors were named according to the predominant food groups, taking into account the authors’ understanding of Zimbabwean dietary practices. The association between these dietary patterns and participant characteristics was assessed using generalised linear models. Initially, univariate analysis for participant characteristics influencing each dietary pattern was performed. These characteristics included alcohol, smoking status, diabetes mellitus, family history of colorectal cancer and cancer, income and level of education. Multivariate analysis was then performed to assess participant characteristics that were independently associated with each dietary pattern. Logistic regression models, adjusted for these significant participant characteristics, were used to determine the association between the different dietary patterns and colorectal cancer. Odds ratios and 95% confidence intervals for the association between the dietary patterns and colorectal cancer were determined. Multinomial logistic regression was used to determine the association between anatomic site and dietary patterns, and relative risk ratios, (with one site as a reference) and 95% confidence intervals computed. All the statistical analysis was performed using Stata MP Version 12.0^®^ (College Station, Texas).

## 3 Results

### 3.1 Characteristics of cases and controls

One hundred cases of colorectal cancer and 200 controls were recruited between November 2012 and December 2015. The tumour location in the cases was as follows: proximal colon 21, distal colon 20, unspecified colon 3, rectum 55, and synchronous colon and rectum 1. The gender distribution and mean age were comparable between cases and controls, and the majority of participants, 71%, lived in urban areas (Table 1). A higher proportion of cases had a tertiary education compared to controls (32% versus 14%, p < 0.001), and more cases earned more than $1000 per month (18% versus 6.5% p = 0.01). The average monthly salary in Zimbabwe is estimated to be $298 per month [18]. Cases were also more likely to have diabetes mellitus, and to have a history of cancer, or colorectal cancer among first degree relatives (Table 1).

**Table 1:** Baseline characteristics of study participants.

Variable	Cases (n = 100)	Controls (n = 200)	P value
Males	50	100	
Mean age, years (SD)	53.1 (14.8)	52.5 (14.6)	0.745
Current residence Rural Urban	29 (29%) 71 (71%)	58 (29%) 142 (71%)	–
Ever drank alcohol	43 (43%)	91 (45.5%)	0.681
Ever smoked	26 (26%)	52 (26%)	1.000
Level of education Primary school Secondary Tertiary	40 (40%) 28 (28%) 32 (32%)	76 (38%) 96 (48%) 28 (14%)	< 0.001
Income (USD) <200 201–500 501–1000 >1000	39 (39%) 29 (29%) 14 (14%) 18 (18%)	75 (37.5%) 83 (41.5%) 29 (14.5%) 13 (6.5%)	0.010
Diabetes Mellitus	7 (7%)	9 (4.5%)	0.046
Cancer in 1st degree relatives	19 (19%)	19 (9.5%)	0.046
Colorectal cancer in 1st degree relatives	2 (2%)	0	0.007

### 3.2 Characteristics of the dietary patterns

Table 2 shows the 31 different food groups used for principal component analysis. Three meaningful factors were identified after principal component analysis (Table 3). These three factors accounted for 75.7% of the variability. A 4th factor, contributing 10.1% of the variability, was also evident, but this could not be adequately characterized, and was dropped from subsequent analysis. Factor 1 was characterised by indigenous Zimbabwean fruits and vegetables, grains, starchy tubers, wild animals and insects, nuts, seeds, and sugarcane. This factor is similar to the traditional Zimbabwean diet, and was named ‘traditional African diet’. Factor 2 was characterised by milk, margarine, potatoes, starches, eggs, poultry, red meat, drinks and beverages. This roughly mirrors the contemporary diet largely consumed in urban areas and was termed ‘urbanised diet’. Factor 3 was characterised by cheese, yoghurt, processed meats, high caloric drinks and snacks, and was termed ‘processed food diet’.

**Table 2:** Food groups according to the FAO dietary diversity score and common culinary usage in Zimbabwe.

Category	Food components
Milk	Lacto (sour milk), full cream milk, whole milk, skimmed milk, powdered milk
Other dairy products	Cheese, yogurt,
Margarine and butter	Margarine and butter
Orange fleshed fruit	mangoes, pawpaw,
Indigenous orange fleshed fruit	chakata, mazhanje
Other fruits	Banana, guava, naartjie, apple, orange, lemon, grape, peaches, pineapple, strawberries,
Indigenous other fruits	Baobab, matohwe
Leafy vegetables	Green vegetables, cabbage (cooked), cabbage (fresh)
Indigenous green leafy vegetables	Munyevhe, muboora, mushamba, mufushwa
Traditional non-leafy vegetables	Okra/Derere,
Orange fleshed crops	Carrot, pumpkin, gourd,
Other non-leafy vegetables	cucumber, pepper, mushrooms/hwowa, salad
Traditional starchy tubers	Sweet potatoes, cassava
Potatoes	Potatoes
Indigenous grains (starch)	Sadza (maize, millet, or sorghum), mealie, porridge(maize)
Starches	Bread (white or dark), biscuits, white rice (cooked), pasta, pizza, samosa, corn flakes/bran flakes, buns/pastries
Eggs	Eggs
Poultry	Chicken, turkey
Wild animals and insects	birds, mice, rabbit, madora, locusts, termites
Red meats	beef, pork, goat or lamb,
Fish	Fish, canned tuna fish
Traditional fish	Matemba (dried fish)
Processed meats	Bacon, ham, corned meat, polony, sausages, burgers, meat pies/sausage roll
Legumes	Beans or lentils, baked beans, peas, soya
Peanuts	Peanuts, peanut butter
Nuts and seeds	Seeds, avocado,
Traditional beverages	Nhopi, maheu
Beverages	Tea and coffee
Drinks	Cascade, orange juice, sugary sweetened soft drinks/freezits, artificial sweetened soft drinks (light)
Snacks	Honey, Candy, Chocolate, ice cream, potato crisps,
Sugar cane	Sugar cane

**Table 3:** Rotated factor loadings.

Variable	Factor 1 (Traditional African diet)	Factor 2 (Urbanised diet)	Factor 3 (Processed food diet)	Uniqueness
Milk	–	0.4657	–	0.71219
Other dairy products			0.6631	0.5504
Margarine and Butter		0.3942		0.7976
Orange fleshed fruit	0.5946			0.6257
Indigenous orange fleshed fruits	0.5791			0.6300
Other Fruit	0.5612			0.6080
Indigenous other fruits	0.4832			0.6885
Leafy vegetables				0.9053
Indigenous green leafy vegetables	0.5485			0.6901
Orange fleshed crops				0.8132
Other non-leafy vegetables	0.3579		0.3741	0.7262
Traditional starchy tubers	0.5101			0.7125
Potatoes		0.4645		0.7639
Indigenous grains (starch)	0.4558			0.7268
Starches		0.7387		0.4133
Eggs		0.4280		0.7134
Poultry		0.4872		0.7592
Wild animals and insects	0.3520			0.7863
Red meat		0.4973		0.7518
Fish				0.9575
Traditional fish (dried fish)				0.9828
Processed meats			0.6357	0.5497
Peanuts				0.8583
Traditional beverages				0.9007
Beverages		0.4603		0.7706
Drinks		0.4619	0.4049	0.6227
Snacks			0.4464	0.7761
Nuts and seeds	0.3920			0.8220
Sugarcane	0.3789			0.8466

Absent scores represent factor loadings <0.35. Kaiser-Meyer-Olkin measure of sampling adequacy 0.7185.

### 3.3 Relationship between dietary patterns and participant characteristics

The univariate analysis for the relationship between each dietary pattern and participant characteristics are shown in supplementary tables. Table 4 shows the three multivariate analyses of the relationship between each dietary pattern and participant characteristics. The traditional African diet was associated with living in rural areas, (OR 1.26; 95%, CI 1.00–1.59), and with a low income compared to the highest income bracket (OR 1.48; 95% CI, 1.06–2.08). There was no association between the consumption of a traditional African diet and education. The urbanised diet was associated with living in urban areas (OR 1.70; 95% CI, 1.38–2.10), and with a secondary (OR 1.30; 95% CI, 1.07–1.59) or a tertiary education (OR 1.48; 95% CI, 1.11–1.97). This urban dietary pattern was associated with an income of $201–500 (OR 1.30; 95% CI, 1.05–1.62), but not higher incomes. The processed food pattern was more likely to be consumed by participants with a tertiary education (OR 1.42; 95% CI, 1.05–1.92), and those who earned more than $1000 per month (OR 1.48; 95% CI, 1.02–2.15). None of the three dietary patterns were associated with diabetes mellitus, smoking, alcohol use or a history of cancer or colorectal cancer in first degree relatives.

**Table 4:** Three multi-variable models for each dietary pattern showing the association between these patterns, and demographic, lifestyle, and socio-economic characteristics.

Variable	Odds ratio	95% CI	P value
Traditional Dietary Pattern			
Colorectal cancer	0.66	0.54–0.80	<0.001
Rural residence	1.26	1.00–1.59	0.046
Income (USD) <200 201–500 501–1000	1.48 1.20 1.24	1.06–2.08 0.87–1.65 0.86–1.79	0.023 0.268 0.260
Urbanised Dietary Pattern			
Colorectal cancer	0.84	0.70–1.00	0.049
Urban residence	1.70	1.38–2.10	<0.001
Never smoked	1.19	0.99–1.43	0.067
Education Secondary Tertiary	1.30 1.48	1.07–1.59 1.11–1.97	0.010 0.007
Income (USD) 201–500 501–1000 >1000	1.30 1.28 1.07	1.05–1.62 0.95–1.71 0.75–1.54	0.018 0.104 0.705
Processed Foods Dietary Pattern			
Colorectal Cancer	0.88	0.76–1.06	0.165
Education Secondary Tertiary	1.20 1.42	0.98–1.48 1.05–1.92	0.085 0.022
Income (USD) 201–500 501–1000 >1000	0.97 1.25 1.48	0.78–1.20 0.93–1.67 1.02–2.15	0.744 0.133 0.038

NB Parameters not appearing on some of the dietary patterns were not significant on univariate analysis.

### 3.4 Relationship between dietary patterns and colorectal cancer

On unadjusted conditional logistic regression, the traditional African dietary pattern was associated with a reduced likelihood of colorectal cancer (OR 0.42; 95% CI, 0.27 – 0.64). This apparently protective association remained after adjusting for income, education and family history (OR 0.35; 95% CI, 0.21 – 0.58) (Table 5). Neither the urbanised (OR 0.68; 95% CI, 0.43–1.08), nor the processed food pattern (OR 0.91; 0.58–1.41) was associated with colorectal cancer, even after adjusting for income, education and family history of cancer (Table 5). There was no association between any of the three dietary patterns with colorectal cancer anatomical sub-sites after adjusting for age (Table 6).

**Table 5:** The relationship between dietary patterns and colorectal cancer adjusted for demographic, socio-economic and clinical characteristics.

Dietary Pattern	Odds Ratio	95% CI	P value
Traditional	0.35	0.21–0.58	<0.001
Urbanised	0.68	0.43–1.08	0.102
Processed	0.91	0.58–1.41	0.659

Income, education, and cancer in 1st degree relatives were potential confounders, and were included in this model.

**Table 6:** The association between dietary patterns and colorectal cancer anatomical subsite, with cancers located in the distal colon as the reference group.

Dietary Pattern	Relative Risk Ratio	95% CI	P value
**Proximal Colon (n = 21)**			
Traditional	3.30	0.75–14.5	0.114
Urbanised	0.40	0.10–1.61	0.196
Processed foods	3.21	0.56–18.48	0.192

**Rectum (n = 55)**			
Traditional	2.37	0.59–9.53	0.226
Urbanised	0.29	0.08–1.00	0.050
Processed food	2.94	0.56–15.44	0.201

Adjusted for age, sex and current residence.

## 4 Discussion

This study confirms that a traditional African dietary pattern, which is predominantly plant and grain based, is associated with a reduced risk of colorectal cancer. While ecological studies suggested that African diets are protective as far back as the 1960s [19], this has never been assessed in a case-control or cohort study [10]. In our study, the traditional African diet was associated with living in rural areas and a low income. The other two dietary patterns, termed urbanised and processed, had no significant effect on colorectal cancer risk, despite high loadings of red meat, and processed meat. This implies that loss of the protective effect of the traditional diets is a more critical determinant of colorectal cancer risk in our population than adoption of potentially adverse ones. It can be speculated that maintaining some components of the traditional African diet may ameliorate the increased risk inherent in adopting modern style diets. Thus differences in the degree of retention of traditional diets may partly explain the variation in colorectal cancer risk described across different countries in sub-Saharan Africa [10].

To an extent, our findings are consistent with previous studies on the effect of dietary patterns on colorectal cancer risk described in other populations. A meta-analysis of these studies concluded that dietary patterns with a high consumption of fruits and vegetables are associated with a reduced risk of colorectal cancer [20]. However, unlike our findings, dietary patterns with a high loading of red meat were associated with an increased risk of colorectal cancer in this meta-analysis. In our study, the traditional dietary pattern is associated with living in rural areas, and a low income. It can be anticipated that dietary transitions will continue with increasing urbanisation, and rising incomes, with a resultant loss of the protective benefits of the traditional African diet. Thus the rising incidence of colorectal cancer can be expected to accelerate in the coming decades, worsening the epidemic of non-communicable diseases in sub-Saharan Africa.

It has been proposed that traditional African diets affect colorectal cancer risk through changing the composition and function of the gut microbiota [21,22]. In an elegant study, swapping the diets of rural South Africans and African-Americans resulted in rapid reciprocal changes in the composition and function of the gut microbiota, and in markers of colonic mucosal proliferation [21]. There was a reduction in butyrate production, with increases in secondary bile acids and colonic mucosal proliferation in Africans commenced on a high fat, low fibre American diet. In contrast, African-Americans commenced on a high fibre, low fat African diet had a rapid increase in butyrate production, with reduction in secondary bile acids and epithelial proliferation. This suggests that the traditional African diets changes gut microbiota function and composition towards production of metabolites favourable to colonic health whilst suppressing potentially carcinogenic ones. A major limitation of this particular study was the use of epithelial proliferation rather than colorectal neoplasia as the study end-point. Our study provides complementary evidence that traditional African diets may be protective of colorectal cancer.

Our study had some limitations which must be taken into account when interpreting the findings. Firstly, the food frequency questionnaire was semi-quantitative, and we did not analyse the data according to the nutrient or absolute food quantity. In addition, familial colorectal cancer syndromes were not excluded, because they are not adequately investigated in routine clinical practice in our setting. However, these generally constitute a small proportion of all colorectal cancer cases (5%), and the effect on the results is likely to be minimal [23]. We further minimised the effect of familial colorectal cancer syndromes by adjusting for family history of cancer during analysis. Moreover, environmental factors such as diet influence the development of colorectal cancer even in familial cancer syndromes [24]. Case-control studies of this nature are also susceptible to differential recall between cases and controls. We tried to minimise this by recruiting cases as soon as possible after diagnosis, and at most within 6 months. We did not collect data on physical activity and body mass index (BMI), as we felt these parameters are particularly prone to differential recall in our setting. Unlike controls, cases would have been asked for a subjective estimate of their pre-morbid weight and physical activity. It is possible that this was the confounder for the association between tertiary education and colorectal cancer in this study. It is reasonable to assume those individuals with a tertiary education were more sedentary and had a higher BMI, which increased their colorectal cancer risk. It may be argued that the association with education suggests differential access to healthcare, but there was no evidence for this during recruitment. Finally, although we achieved the desired sample size, the study can still be regarded as small in comparison to similar studies, and this may have masked the potentially deleterious impact of the urban and processed foods patterns.

In conclusion, our findings re-affirms the protective properties of the traditional African diets, and demonstrate that urbanisation and rising incomes are associated with a shift from these protective diets. These findings provide a basis for designing primary intervention strategies for populations undergoing dietary transitions, and adds to the general evidence base on the role of diet in colorectal cancer. The promotion of traditional diets in these populations may slow the rise in colorectal cancer. Moreover, such interventions may have a salutary effect on other non-communicable diseases in particular obesity and diabetes mellitus, which to an extent, have similar risk factors to colorectal cancer [25,26]. This may be vital in sub-Saharan Africa, where strategies based on screening are unlikely to be feasible or cost-effective.

## Authorship contribution statement

Leolin Katsidzira: Contributed to conception and design, acquisition of data, analysis and interpretation of data, drafting the article and final approval of the submitted version.

Ria Laubscher: Contributed to analysis and interpretation of data, revising the article critically for important intellectual content and final approval of the submitted version.

Innocent T Gangaidzo: Contributed to conception and design, acquisition of data, and analysis and interpretation of data, revising the article critically for important intellectual content and final approval of the submitted version.

Rina Swart: Contributed to analysis and interpretation of data, revising the article critically for important intellectual content and final approval of the submitted version.

Rudo Makunike-Mutasa: Contributed to acquisition of data, analysis and interpretation of data, revising the article critically for important intellectual content and final approval of the submitted version.

Tadios Manyanga: Contributed to acquisition of data, revising the article critically for important intellectual content and final approval of the submitted version

Sandie Thomson: Contributed to analysis and interpretation of data, revising the article for important intellectual content and final approval of the submitted version.

Raj Ramesar: Contributed to conception and design, analysis and interpretation of data, revising the article critically for important intellectual content and final approval of the submitted version.

Jonathan A Matenga: Contributed to conception and design, analysis and interpretation of data, revising the article critically for important intellectual content and final approval of the submitted version.

Simbarashe Rusakaniko: Contributed to conception and design, acquisition of data, and analysis and interpretation of data, revising the article critically for important intellectual content and final approval of the submitted version.

## Declarations of interest

None.
