# Supplementary material for: Dietary patterns and colorectal cancer risk in Zimbabwe: A population based case-control study
Source: Cancer Epidemiol. 2018 Dec;57:33–8. doi: 10.1016/j.canep.2018.09.005 (PMC6291434; doi:10.1016/j.canep.2018.09.005)
Supplement: Supplementary file 1 [file mmc1.docx]

**Supplementary Material**

**Supplementary Table 1. Univariate analysis of the association between the traditional dietary pattern, and demographic, socio-economic, and clinical characteristics**

| Variable | Odds ratio | 95% CI | P value |
| --- | --- | --- | --- |
| Colorectal cancer | 0.65 | 0.61 – 0.79 | <0.001 |
| Urban residence | 0.78 | 0.61 – 0.98 | 0.035 |
| Ever drank alcohol | 1.07 | 0.85 – 0.74 | 0.552 |
| Ever smoked | 0.98 | 0.75 – 1.27 | 0.880 |
| Education  Secondary  Tertiary | 1.08  1.19 | 0.86 – 1.35  0.86 – 1.65 | 0.533  0.287 |
| Income (USD)  201 - 500  501 – 1000  >1000 | 0.80  0.80  0.60 | 0.63 – 1.03  0.58 – 1.12  0.39 – 0.90 | 0.080  0.199  0.015 |
| Diabetes mellitus | 0.82 | 0.54 – 1.24 | 0.353 |
| Cancer in 1^st^ degree relatives | 1.01 | 0.76 – 1.35 | 0.928 |
| Colorectal cancer in 1^st^ degree relatives | 1.03 | 0.31 – 3.37 | 0.964 |

**Supplementary Table 2. The association between the urbanised dietary pattern, and demographic, socio-economic, and clinical characteristics**

| Variable | Odds Ratio | 95% CI | P value |
| --- | --- | --- | --- |
| Colorectal cancer | 0.83 | 0.69 – 0.99 | 0.043 |
| Urban residence | 1.69 | 1.37 – 2.09 | <0.001 |
| History of alcohol use | 1.09 | 0.88 – 1.34 | 0.438 |
| Ever smoked | 0.79 | 0.63 – 1.00 | 0.050 |
| Education  Secondary  Tertiary | 1.26  1.41 | 1.03 – 1.55  1.05 – 1.89 | 0.023  0.021 |
| Income  201 – 500  501 – 1000  >1000 | 1.33  1.30  1.13 | 1.07 – 1.66  0.97 – 1.75  0.78 – 1.64 | 0.011  0.080  0.529 |
| Diabetes Mellitus | 0.93 | 0.64 – 1.35 | 0.700 |
| Cancer in 1^st^ degree relatives | 0.99 | 0.76 – 1.28 | 0.930 |
| Colorectal cancer in 1^st^ degree relatives | 0.74 | 0.26 – 2.15 | 0.586 |

**Supplementary Table 3. The association between the processed food pattern, and demographic, socio-economic, and clinical characteristics**

| Variable | Odds ratio | 95% CI | P value |
| --- | --- | --- | --- |
| Colorectal cancer | 0.87 | 0.71 – 1.05 | 0.149 |
| Urban residence | 1.09 | 0.87 – 1.36 | 0.456 |
| Ever used alcohol | 1.13 | 0.91 – 1.41 | 0.259 |
| Ever smoked | 0.96 | 0.75 – 1.24 | 0.776 |
| Education  Secondary  Tertiary | 1.15  1.38 | 0.93 – 1.43  1.01 – 1.87 | 0.200  0.042 |
| Income (USD)  201 – 500  501 – 1000  >1000 | 0.96  1.23  1.48 | 0.76 – 1.43  0.90 – 1.68  1.00 – 2.20 | 0.760  0.194  0.051 |
| Diabetes Mellitus | 0.81 | 0.55 – 1.20 | 0.286 |
| Cancer in 1^st^ degree relatives | 0.97 | 0.74 – 1.27 | 0.818 |
| Colorectal cancer in 1^st^ degree relatives | 0.65 | 0.21 – 2.01 | 0.458 |
